# Glutamate decarboxylase 1 (GAD1) suppresses the progression of glioblastoma through GSK3β/β-catenin pathway

**DOI:** 10.1038/s41420-026-02997-0

**Published:** 2026-03-17

**Authors:** Yanwen Zheng, Zhaomin Zhong, Chiyu Zhang, Jin Gu

**Affiliations:** 1 Central Laboratory, The Second Affiliated Hospital of Soochow University, Suzhou, China; 2 School of Basic Medical Sciences, Suzhou Medical College, Soochow University, Suzhou, China

**Keywords:** Oncogenes, CNS cancer, Cell growth

## Abstract

Glioblastoma multiforme (GBM) is the most common and aggressive primary brain tumor. Glutamate decarboxylase 1 (GAD1), which mainly produces gamma-aminobutyric acid (GABA) in neurons, has also been implicated in tumor progression. However, the role of GAD1 in glioma is not well understood. Our study found that GAD1 expression is downregulated in glioma, correlating with poor prognosis in glioma patients. Overexpression of GAD1 suppressed glioblastoma cell proliferation, colony formation, cell cycle progression, migration, and invasion, whereas knockdown of GAD1 promoted these phenotypes. Furthermore, GAD1 overexpression significantly reduced the protein levels of p-GSK3β (ser9) and β-catenin, as well as the downstream molecules Cyclin D1 and MMP9, whereas GAD1 knockdown increased their expression. The GSK3β inhibitor AR-A014418 effectively counteracted the effects of GAD1 knockdown, suppressing the enhanced proliferation, cell cycle progression, and invasion of glioblastoma cells, while also reducing the expression of p-GSK3β, β-catenin, Cyclin D1, and MMP9. Furthermore, zebrafish xenotransplantation experiments demonstrated that GAD1 overexpression suppressed tumor growth, whereas GAD1 knockdown facilitated tumor formation. Collectively, these results suggest that GAD1 inhibits the expression of Cyclin D1 and MMP9 via the p-GSK3β/β-catenin pathway, thereby impeding glioblastoma progression. These findings may offer a novel therapeutic target for glioblastoma treatment.

## Introduction

Glioma is a primary malignant neoplasm of the central nervous system, originating from glial cells within the brain. Among these, glioblastoma multiforme (GBM) is recognized as the most prevalent and lethal variant of brain cancer [1, 2]. Despite significant advancements in surgical interventions and pharmacological treatments, the prognosis for glioblastoma patients remains dismal, with a median survival duration of approximately 15 months [3, 4]. The diffuse growth pattern characteristic of glioblastoma significantly impedes curative surgical resection [5, 6]. Consequently, identification of novel therapeutic targets and prognostic markers for glioma is of critical importance.

Glutamate decarboxylase (GAD) catalyzes the decarboxylation reaction of glutamate to produce gamma-aminobutyric acid (GABA) [7]. The GABAergic neurons in the mammalian nervous system express two homologous isoforms of GAD: the 67-kDa and 65-kDa variants (GAD67 and GAD65), encoded by the GAD1 and GAD2 genes, respectively [8, 9]. GAD1 predominantly contributes to the basal synthesis of GABA in neuronal cells, and *Gad1* knockout mice exhibit cleft palate and perinatal lethality [10, 11]. Recent research indicates that GAD1 is critically involved in the pathogenesis of various tumors. Analyses of lung adenocarcinoma samples and databases have revealed that the mRNA expression level of GAD1 is elevated in lung cancer patient samples compared to normal tissues. Furthermore, elevated GAD1 levels correlate with poorer prognosis and reduced survival periods [12]. Studies have indicated that GAD1 expression is upregulated in oral squamous cell carcinoma cells relative to normal oral keratinocytes. Suppression of GAD1 expression resulted in the inhibition of β-catenin translocation and MMP7 secretion, thereby significantly diminishing the invasive and migratory capacities of cancer cells [13]. Additionally, another study reported a marked increase in GAD1 expression levels in both benign and malignant prostate cancer tissues [14].

However, research on GAD1 in glioma remains notably limited. An analysis of the GBM database demonstrated that the expression levels of three GABA-related genes-GAD1, GAD2, and 4-aminobutyrate aminotransferase (ABAT)-were reduced in mesenchymal tumors [15]. Other studies using deep learning have identified the top ten genes associated with glioblastoma stem cells, the stem cell microenvironment, and treatment resistance mechanisms, among which GAD1 is included [16].

In recent years, the zebrafish (Danio rerio) has emerged as a highly valuable model organism for xenotransplantation research [17–19]. The transparency of zebrafish embryos and larvae permits the visualization of internal structures, including organs. Additionally, the in vitro development facilitates microinjection and enables real-time observation of tumor cell proliferation post-microinjection [18].

In this study, we initially identified that the expression of GAD1 is downregulated in glioblastoma. Subsequently, we demonstrated that overexpression of GAD1 significantly inhibits the proliferation, colony formation, cell cycle progression, migration, and invasion of glioblastoma cells, whereas knockdown of GAD1 produces contrary effects. Furthermore, our findings indicate that GAD1 suppresses the p-GSK3β/β-catenin signaling pathway, leading to a reduction in the expression of Cyclin D1 and MMP9, thereby inhibiting the progression of glioblastoma. Additionally, we established a xenograft model by microinjecting human glioblastoma cells into zebrafish larvae. In this model, we observed that GAD1 overexpression effectively impedes tumor growth in zebrafish.

## Results

### GAD1 is downregulated in glioma and predicts a poor prognosis

**Fig. 1:**
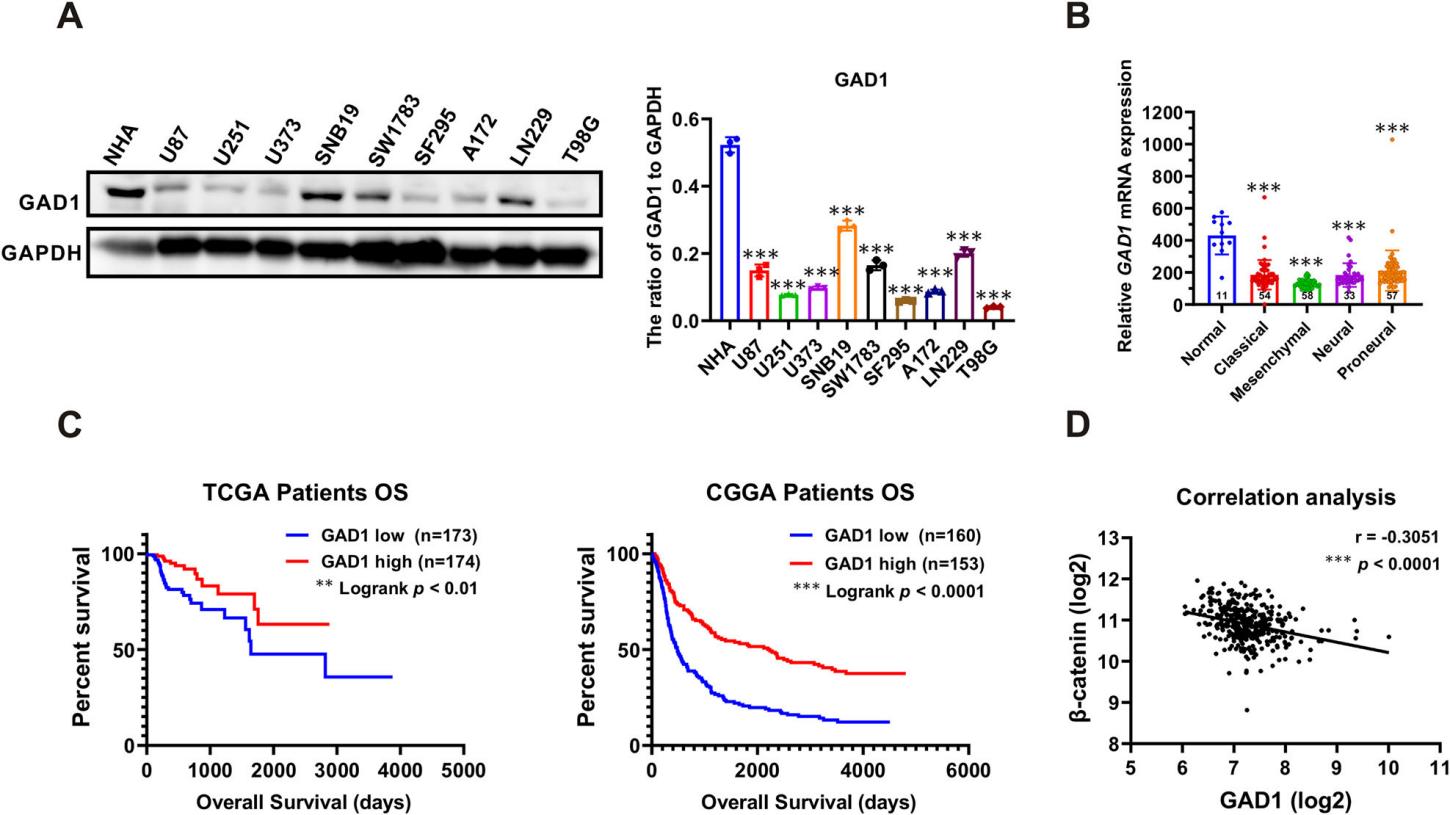
GAD1 is downregulated in glioma and is associated with a poor prognosis. **A** Western blot analysis was conducted to assess GAD1 expression in human glioma cell lines and immortalized normal astrocytes (NHA), with GAPDH serving as a loading control. **B** TCGA dataset analysis of the GAD1 mRNA expression levels in GBM subtypes and normal brain tissues. ***p < 0.001, compared with normal. Normal: *n* = 11; Classical: *n* = 54; Mesenchymal: *n* = 58; Neural: *n* = 33; Proneural: *n* = 57. **C** Kaplan–Meier survival analysis was performed to evaluate the overall survival (OS) of glioma patients with low or high GAD1 expression in both the TCGA (left) and CGGA (right) databases. The median expression level was used as a cutoff point to differentiate between patients with low and high GAD1 expression. **D** Pearson correlation of GAD1 expression with β-catenin in GBM patient samples (*n* = 356).

We first examined the protein expression levels of GAD1 in glioma cell lines by Western blot. The analysis revealed a significant decrease in GAD1 levels across all examined glioma cell lines compared to normal human astrocytes (NHA) (Figs. 1A, S1). Analysis of the Cancer Genome Atlas (TCGA) dataset showed reduced GAD1 mRNA levels across all four glioblastoma subtypes, with the lowest expression in the mesenchymal subtype (Fig. 1B). Data from TCGA and the Chinese Glioma Genome Atlas (CGGA) indicated that low GAD1 levels correlated with poor prognosis in glioma (Fig. 1C). Furthermore, a significant negative correlation was observed between β-catenin and GAD1 expression (Pearson’s *r* = −0.3051, *p* < 0.0001) (Fig. 1D). The results indicate that decreased GAD1 expression in glioma is associated with a poor prognosis.

### Overexpression of GAD1 suppresses the proliferation, colony formation, cell cycle progression, migration and invasion of glioblastoma cells

To explore the role of GAD1 in GBM, we conducted gain-of-function studies in U87 and T98G cell lines (Fig. 1A). These cells were infected with GAD1 or control LV8N lentiviruses and screened with puromycin.

**Fig. 2:**
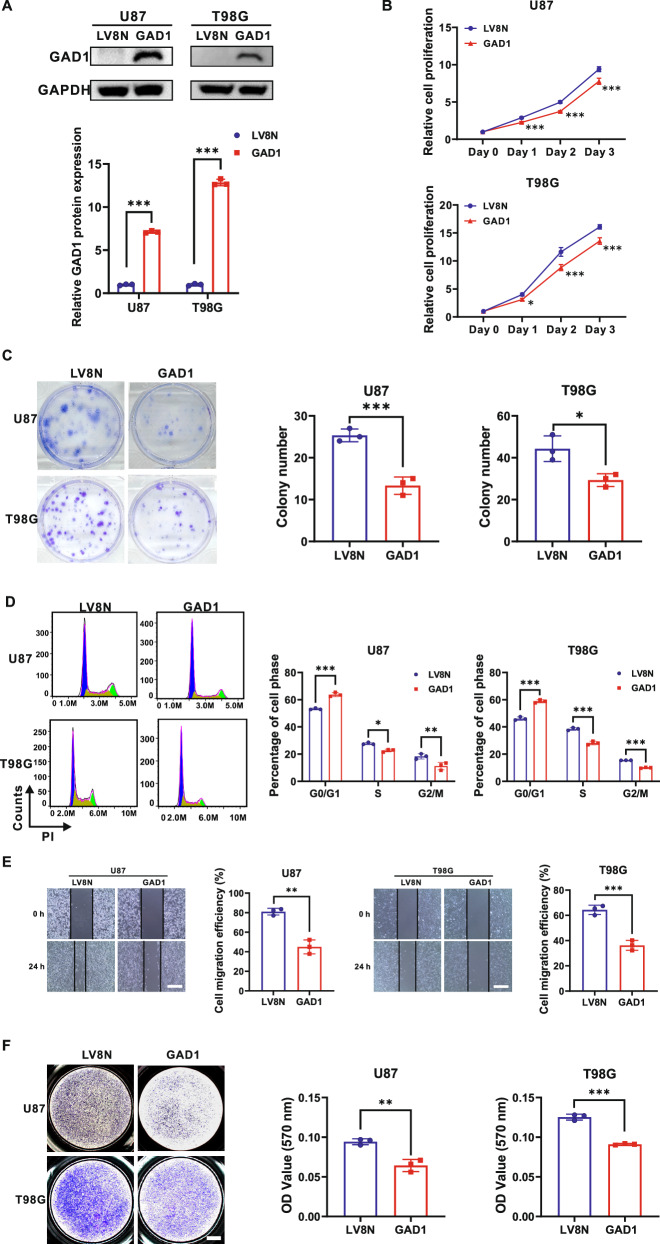
Overexpression of GAD1 suppresses the proliferation, colony formation, cell cycle progression, migration, and invasion of glioblastoma cells. **A** Western blot analysis was performed to examine GAD1 expression in U87 and T98G cells, which were infected with lentiviruses expressing either GAD1 or the control vector LV8N. GAPDH was used as a loading control. **B** The impact of GAD1 overexpression on cell proliferation was assessed in U87 and T98G cells by CCK-8. **C** A colony formation assay was conducted in U87 and T98G cell lines with GAD1 overexpression, and the number of colonies was quantified. **D** The cell cycle analysis of U87 and T98G cells, which infected with LV8N or GAD1 lentiviruses, was conducted using flow cytometry, and the distribution of cells across different phases was depicted in a bar graph. **E** The impact of GAD1 overexpression on the migratory capacity of U87 and T98G cells was assessed through a wound healing assay, with cell migration efficiency calculated as follows: [(wound width at 0 h - wound width at 24 h)/wound width at 0 h] × 100%. **F** The influence of GAD1 on cell invasion was evaluated using the Matrigel Transwell assay, with OD570 serving as an indicator of invasive potential. Scale bar: 400 μm (**E**), 1000 μm (**F**). *p < 0.05, **p < 0.01, ***p < 0.001.

The immunoblotting results indicated a significant upregulation of GAD1 expression in U87 and T98G cells infected with GAD1 lentiviruses (Figs. 2A, S2). The CCK-8 assay demonstrated that GAD1 overexpression significantly inhibited the proliferation of U87 and T98G cells (Fig. 2B). Consistently, the colony formation assay revealed a marked reduction in colony numbers upon GAD1 upregulation in U87 and T98G cells (Fig. 2C). To investigate the mechanism underlying GAD1-mediated inhibition of cell proliferation, cell cycle analysis was conducted. Compared to the control cells infected with LV8N lentiviruses, GAD1 overexpression in U87 and T98G cell lines resulted in a significant increase in the percentage of cells in the G0/G1 phase by approximately 10% (both *p* < 0.001), along with a significant decrease in the percentage of cells in the S (*p* < 0.05, *p* < 0.001, respectively) and G2/M (*p* < 0.01, *p* < 0.001, respectively) phases (Fig. 2D). A wound healing assay was conducted to assess the impact of GAD1 on cell migration. As illustrated in Fig. 2E, the scratch widths in U87 and T98G cells overexpressing GAD1 were noticeably wider than those in the control cells 24 h post-scratch. Upon upregulation of GAD1 expression, the migration efficiency of U87 cells decreased by approximately 35.99% (*p* < 0.01), while that of T98G cells decreased by about 28.14% (*p* < 0.001) (Fig. 2E). An invasion assay utilizing Matrigel and Transwell chambers was performed to evaluate the impact of GAD1 on the invasive capacity of GBM cells. Cells that traversed the membrane were stained with crystal violet, then dissolved in 5% SDS and read by spectrophotometer for OD570. In comparison to the control group, the OD570 values of GAD1-overexpressing cells were significantly reduced in both U87 and T98G cell lines (*p* < 0.01 and *p* < 0.001, respectively) (Fig. 2F). These findings demonstrate that GAD1 overexpression suppresses glioblastoma cell proliferation, colony formation, cell cycle progression, migration, and invasion.

### Down-regulation of GAD1 expression promotes the proliferation, colony formation, cell cycle progression, migration, and invasion of glioblastoma cells

**Fig. 3:**
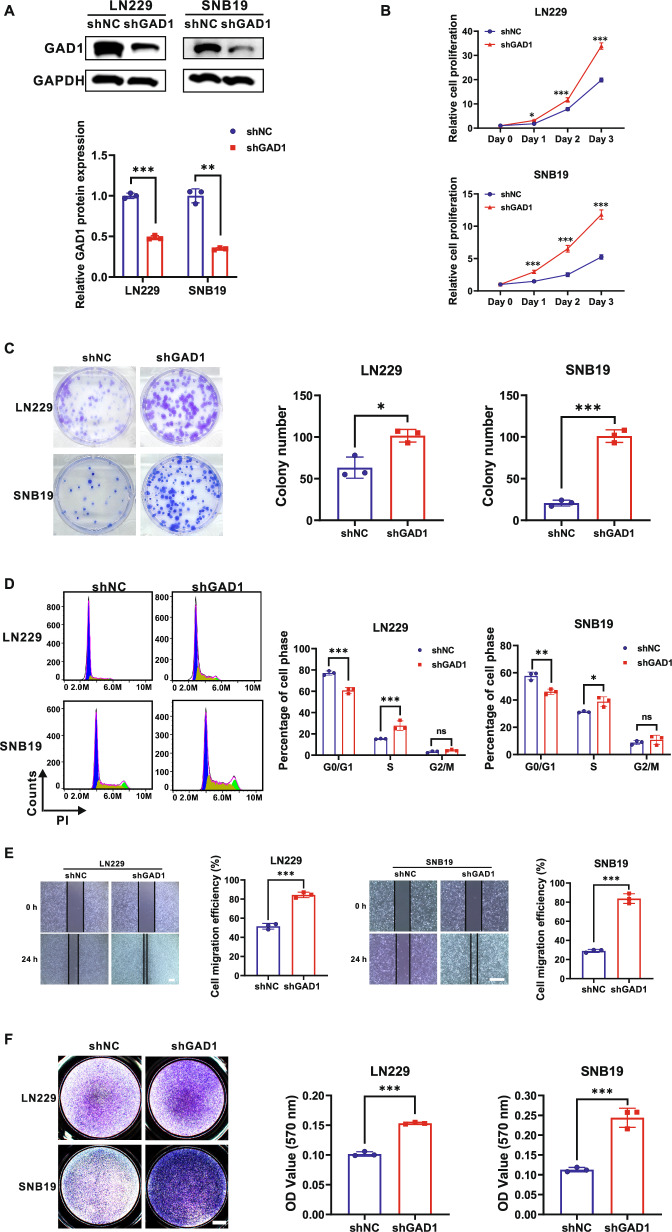
Down-regulation of GAD1 expression promotes the proliferation, colony formation, cell cycle progression, migration, and invasion of glioblastoma cells. **A** Western blot analysis was conducted to detect GAD1 expression in LN229 and SNB19 cells infected with either shNC or shGAD1 lentiviruses, using GAPDH as a loading control. GAD1 knockdown was found to enhance cell proliferation (**B**), colony formation (**C**), cell cycle progression (**D**), cell migration (**E**), and invasion (**F**) in LN229 and SNB19 cells. Scale bar: 400 μm (**E**), 1000 μm (**F**). *p < 0.05, **p < 0.01, ***p < 0.001. ns not significant.

To further substantiate these findings, loss-of-function approaches were employed. The LN229 and SNB19 glioblastoma cell lines were selected for this study (Fig.1A). Western blot analysis demonstrated that, in comparison to the control lentivirus shNC, the lentivirus shGAD1 significantly decreased the GAD1 protein levels in LN229 and SNB19 cells (Figs. 3A, S3). The CCK-8 assay indicated that silencing GAD1 significantly enhanced cell proliferation in both LN229 and SNB19 cells (Fig. 3B). Correspondingly, the number of colonies was markedly increased following GAD1 knockdown in these cell lines (Fig. 3C). Cell cycle analysis revealed that GAD1 knockdown resulted in a reduction in the proportion of cells in the G0/G1 phase (*p* < 0.001, *p* < 0.01, respectively) and an increase in the percentage of cells in the S phase (*p* < 0.001, *p* < 0.05, respectively) in LN229 and SNB19 cells (Fig. 3D). The wound healing assay demonstrated that GAD1 knockdown enhanced cell migration efficiency by approximately 32.51% (*p* < 0.001) in LN229 cells and approximately 54.71% (*p* < 0.001) in SNB19 cells (Fig. 3E). Furthermore, knockdown of GAD1 significantly enhanced the invasion capabilities of LN229 and SNB19 cells (both *p* < 0.001) (Fig. 3F). These findings suggest that down-regulation of GAD1 expression facilitates the proliferation, colony formation, cell cycle progression, migration, and invasion of glioblastoma cells.

### GAD1 modulates the expression of MMP9 and Cyclin D1 through the p-GSK3β/β-catenin signaling pathway in glioblastoma

**Fig. 4:**
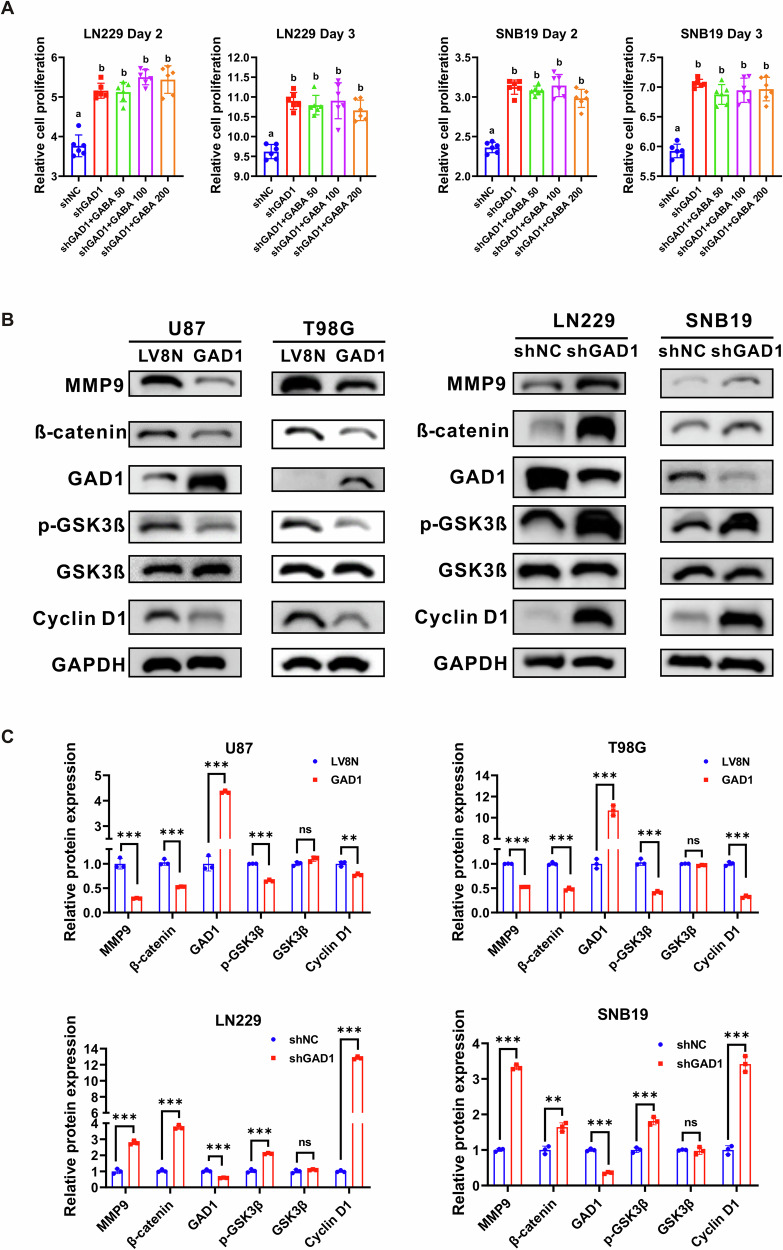
GAD1 modulates the expression of MMP9 and Cyclin D1 via the p-GSK3β/β-catenin signaling pathway in glioblastoma. **A** The impact of varying concentrations of GABA on the proliferation of LN229 and SNB19 cells with GAD1 knockdown. **B**, **C** Western blot analysis was performed to assess the expression levels of MMP9, β-catenin, p-GSK3β, and Cyclin D1 in U87 and T98G cells with GAD1 overexpression, as well as in LN229 and SNB19 cells with GAD1 knockdown, using GAPDH as an endogenous control. Statistical significance was noted with b: *p* < 0.001, compared to a. ** *p* < 0.01, *** *p* < 0.001. ns not significant.

Notably, GAD1 serves a vital function in the nervous system by converting glutamate, an excitatory neurotransmitter, into GABA, the primary inhibitory neurotransmitter in the central nervous system [20, 21]. To evaluate whether the enhanced cell proliferation resulting from GAD1 knockdown could be mitigated, GABA supplementation was administered to LN229 and SNB19 cells following GAD1 knockdown. According to the relevant literature [22, 23], the cells were supplemented with GABA at final concentrations of 50, 100, and 200 μM. Compared to the shNC groups, the proliferation rates in the shGAD1 groups were markedly increased in both LN229 and SNB19 cells. However, varying concentrations of GABA (50, 100, 200 μM) failed to decrease cell proliferation in the shGAD1 groups across both cell lines, irrespective of the time points assessed (day 2 or day 3) (Fig. 4A). The results indicate that the proliferation changes induced by GAD1 levels are not attributable to GABA levels.

In this study, we aimed to elucidate the signaling pathways regulated by GAD1 that are implicated in the pathogenesis of GBM. Glycogen synthase kinase 3β (GSK-3β) is a multifunctional serine/threonine protein kinase that significantly contributes to tumor cell growth, proliferation, and invasion [24–26]. It is well-established that MMP9 is involved in cellular migration and invasion, whereas Cyclin D1 facilitates the transition from the G1 phase to the S phase of the cell cycle. As depicted in Fig. 4B, C, overexpression of GAD1 markedly reduced the protein levels of phosphorylated GSK-3β (p-GSK3β) (ser9) and its downstream effector β-catenin, as well as the β-catenin downstream targets MMP9 and Cyclin D1 in U87 and T98G cell lines. Conversely, down-regulation of GAD1 expression led to an upregulation of p-GSK3β (ser9), β-catenin, MMP9, and Cyclin D1 protein levels in LN229 and SNB19 cells (Figs. 4B, C, S4–S7). These findings indicate that GAD1 modulates the p-GSK3β/β-catenin signaling pathway, thereby influencing the expression of MMP9 and Cyclin D1 in glioblastoma.

### Inhibition of GSK3β counteracts the effects of GAD1 knockdown on proliferation, cell cycle progression, and invasion of glioblastoma cells

**Fig. 5:**
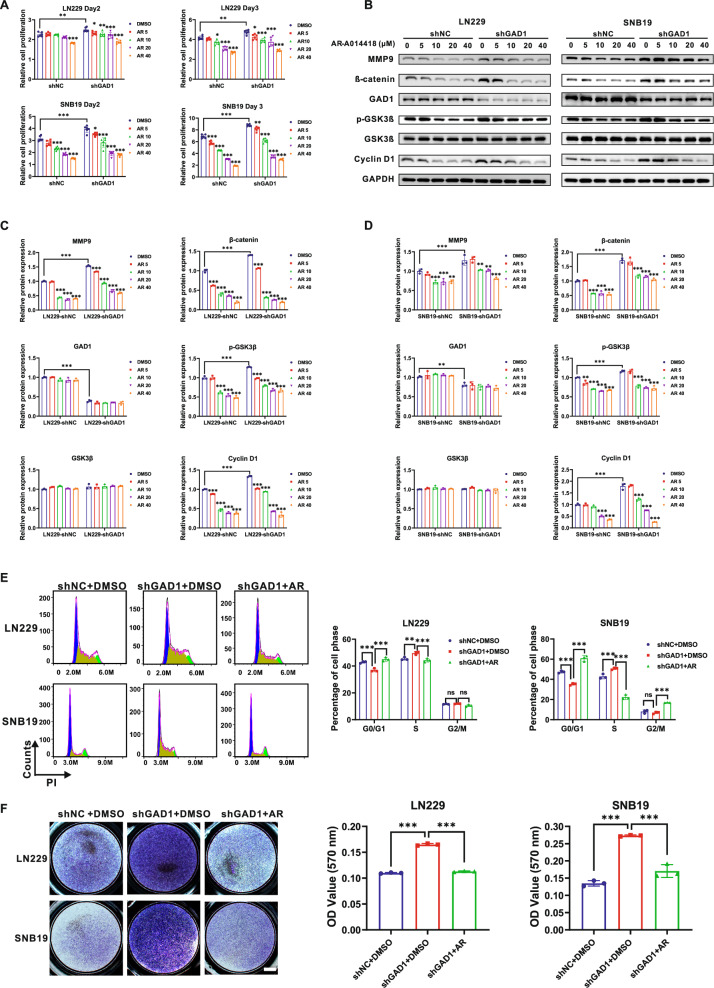
Inhibition of GSK3β counteracts the effects of GAD1 knockdown on proliferation, cell cycle progression, and invasion in glioblastoma cells. **A** CCK-8 assay was conducted to evaluate the influence of AR-A014418, a GSK3β inhibitor, on the proliferation of LN229 and SNB19 cells with GAD1 knockdown. The results, depicted in the figure, indicate relative proliferation on day 2 and day 3. **B**–**D** Treatment of LN229 and SNB19 cells with AR-A014418 for 48 h led to a significant reduction in the expression of MMP9, β-catenin, p-GSK3β, and Cyclin D1 in a dose-dependent manner, with the exception of GAD1, as demonstrated by Western blot analysis. AR-A014418 diminished the GAD1 knockdown-enhanced cell cycle progression (**E**) and cell invasion (**F**) in LN229 and SNB19 cells. Scale bar: 1000 μm (**F**). *p < 0.05, **p < 0.01, ***p < 0.001. ns not significant.

Previous studies have indicated that GSK3β facilitates the survival and proliferation of glioblastoma cells while protecting them from apoptosis [27]. Our findings reveal that GAD1 modulates the expression of p-GSK3β (Fig. 4B), suggesting that GSK3β may play a role in the pathological processes of GBM that regulated by GAD1. To further investigate this, we employed the GSK3β pharmacological inhibitor AR-A014418 at varying concentrations (5, 10, 20, 40 μM) or dimethyl sulfoxide (DMSO) as a vehicle control to treat LN229 and SNB19 cells with or without GAD1 knockdown. Consistent with previous findings, DMSO-treated LN229 and SNB19 cells in the shGAD1 group continued to exhibit enhanced proliferative capabilities compared to those in the shNC group. However, AR-A014418 effectively inhibited the GAD1 knockdown-induced cell proliferation in a concentration-dependent manner, observable on both day 2 and day 3 (Fig. 5A).

Subsequently, we conducted Western blot analyses to assess the expression levels of several key proteins within the cells following 48 h of treatment with AR-A014418. The findings indicated that in both the shNC and shGAD1 groups of LN229 and SNB19 cells, AR-A014418 inhibited the expression of p-GSK3β as well as other proteins associated with proliferation and migration, including β-catenin, MMP9, and Cyclin D1, in a concentration-dependent manner. Notably, the protein level of GAD1 remained unaffected (Figs. 5B–D, S8–S9). To further elucidate the impact of AR-A014418 on GAD1 function, we performed cell cycle analysis and invasion assay. As illustrated in Fig. 5E, treatment with 20 μM AR-A014418 counteracted the reduction in the G0/G1 phase proportion and the increase in the S phase proportion induced by GAD1 knockdown in LN229 and SNB19 cells (Fig. 5E). Similarly, 20 μM AR-A014418 diminished the cell invasion enhanced by GAD1 knockdown in these cell lines (Fig. 5F). Collectively, these results suggest that GAD1 modulates the levels of MMP9 and Cyclin D1 via the p-GSK3β/β-catenin signaling pathway, thereby influencing glioblastoma progression.

### The influence of GAD1 on tumor growth in zebrafish

**Fig. 6:**
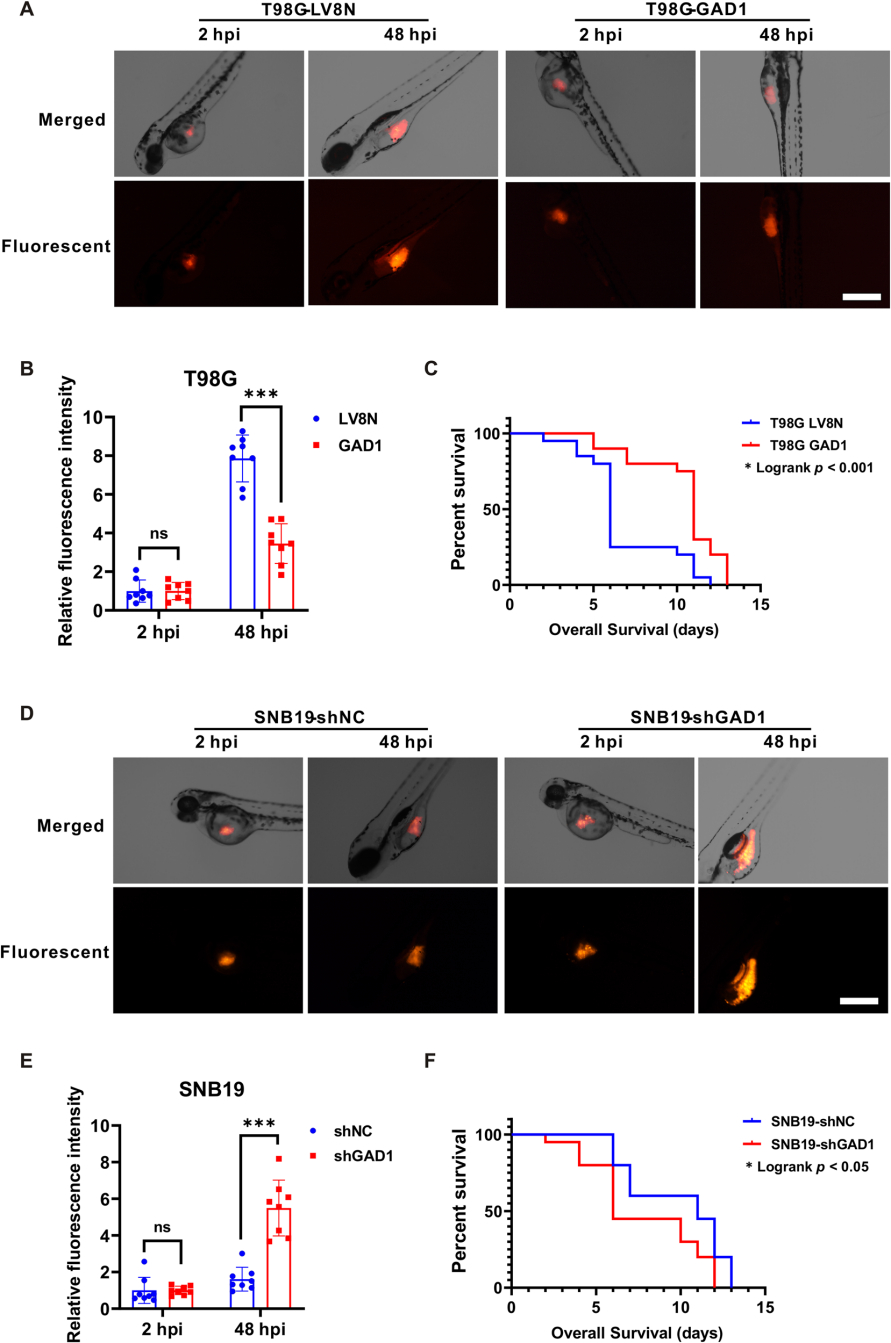
The influence of GAD1 on tumor growth in zebrafish models. Glioblastoma cells, tagged with red fluorescence, were injected into the central yolk sac of zebrafish larvae. Both bright-field and fluorescence imaging were conducted at 2 hpi and 48 hpi. **A** The merged and fluorescent images indicated a noticeable increase in the size of T98G-LV8N tumor masses at 48 hpi, whereas tumors overexpressing GAD1 exhibited only a marginal size increase. **B** At 48 hpi, the relative fluorescence intensity of the T98G-GAD1 tumors was significantly lower than that of control LV8N tumors in zebrafish. **C** A Kaplan–Meier survival analysis was performed on zebrafish bearing T98G-LV8N or T98G-GAD1 tumors (20 zebrafish per group), with statistical evaluation via a log-rank test, *p* < 0.001. **D** The SNB19-shGAD1 tumors, with GAD1 knockdown, demonstrated a significantly larger size than the shNC control group at 48 hpi. **E** The relative fluorescence intensity of SNB19-shGAD1 tumors was markedly higher than that of shNC tumors at 48 hpi. **F** The survival analysis of zebrafish with SNB19-shNC or SNB19-shGAD1 tumors was conducted (20 zebrafish per group), *p* < 0.05.

Zebrafish is an ideal model for xenotransplantation. This study examined the function of GAD1 in zebrafish bearing tumors. Images were captured at 2 hpi and 48 hpi following the microinjection of glioblastoma cells labeled with red fluorescence into the central yolk sac of zebrafish, revealing that the injected cells accumulated within the larvae. After 48 h of growth, the size of the T98G-LV8N tumors in the control group exhibited a significant increase, whereas the tumors in the T98G-GAD1 group remained comparatively smaller (Fig. 6A). The fluorescence emitted by each group within the larvae was quantified using ImageJ software to assess the in vivo proliferation of glioblastoma cells. At 48 hpi, the relative fluorescence intensity in the zebrafish of the GAD1 group was significantly lower than that in the control group (Fig. 6B). Additionally, we investigated the proliferation of GAD1-knockdown glioblastoma cells in zebrafish. At 48 hpi, the tumor size in the SNB19-shGAD1 group was significantly larger than that in the shNC group (Fig. 6D), and the relative fluorescence intensity in the shGAD1 group was also markedly higher than that in the control group (Fig. 6E). Survival studies have indicated that overexpression of GAD1 significantly extended the survival duration of zebrafish with tumors (*p* < 0.001) (Fig. 6C), whereas knockdown of GAD1 reduced the survival period (*p* < 0.05) (Fig. 6F). The in vivo experiments revealed that GAD1 overexpression inhibits tumor growth, while GAD1 knockdown facilitates tumor progression in zebrafish.

## Discussion

Glioblastoma cannot be fully removed due to their diffuse growth, which makes the search for new diagnostic markers and therapeutic targets crucial. This study showed that decreased GAD1 levels in glioma correlate with lower patient survival rates. Overexpression of GAD1 inhibits glioblastoma cell proliferation, colony formation, cell cycle progression, migration, and invasion, and reduces tumor growth in a zebrafish model. Conversely, down-regulation of GAD1 expression has the opposite effect. GAD1 influences glioblastoma progression by regulating MMP9 and Cyclin D1 via the p-GSK3β/β-catenin pathway.

GAD1 is ubiquitously expressed throughout the cell and is primarily responsible for GABA synthesis [20, 21]. Research indicates that low GAD1 gene expression in the prefrontal cortex and hippocampus is common in schizophrenia and bipolar disorder patients [28–30]. GAD 1 methylation and mRNA levels are higher in lung adenocarcinoma (LADC) than in non-tumor tissues, correlating with poorer survival outcomes [12]. In mice with transplanted renal cancer cells, miR-4284 targeted GAD1, inhibited the expression of proliferation-related gene PCNA, and promoted the expression of pro-apoptotic gene BAX [31].

However, studies on the role of GAD1 in glioma are limited. Some researchers have identified eight promoter-hypermethylated genes in gliomas, including GAD1. Abnormal promoter methylation and altered histone modifications are associated with the decreased expression of these genes [32]. A study found that GAD1 overexpression enhances the sensitivity of glioma cells to cuproptosis through the RAS/MAPK pathway [33]. In this study, we detected a significant decrease of GAD1 protein in glioma cell lines (Fig. 1A). Data from TCGA revealed that GAD1 mRNA levels were significantly lower in all four GBM subtypes compared to normal tissues. (Fig. 1B), and the expression levels of GAD1 were positively correlated with the survival period of glioma patients (Fig. 1C). Functional analysis showed that GAD1 overexpression inhibited cell proliferation, colony formation, cell cycle progression, migration, and invasion in U87 and T98G cells (Fig. 2), while GAD1 knockdown had the opposite effect in LN229 and SNB19 cells (Fig. 3). Our findings suggest that GAD1 mainly inhibits glioblastoma progression.

Glutamine can be converted by GAD1 into the non-protein amino acid GABA, which plays a major role as an inhibitory neurotransmitter in the mammalian central nervous system [34]. Apart from the nervous system, elevated GABA levels are linked to cancer progression in several tumors, such as colon cancer, breast cancer, prostate cancer and gastric cancer [35–38]. A Study has reported that 100 μM GABA promoted the proliferation of lung cancer cell line H520 and colorectal cancer cell line HT29 with GAD1 knockdown, while exerting no effect on control cells [22]. Another study reported that 100 μM of GABA can bind to GABA_B_ receptors to inhibit the proliferation of cholangiocarcinoma cell line QBC939, possibly through the JAK/STAT3 pathway [23]. This study explored whether GAD1’s impact on glioblastoma was due to GABA synthesis. Surprisingly, varying concentrations of GABA did not counteract the increased cell proliferation induced by GAD1 knockdown (Fig. 4A), indicating GAD1 affects glioblastoma progression through mechanisms other than GABA.

GSK-3β is a serine-threonine kinase involved in processes such as proliferation, metastasis, invasion, and apoptosis in tumors, including glioma [24–26]. Inhibiting GSK-3β reduced the proportion of stem cell-like CD133+ cells of LN319 glioblastoma cells and decreased the expression of SOX2 in the isolated neural spheres [26]. Phosphorylation of tyrosine 216 and serine 9 respectively activates or inhibits the kinase activity of GSK-3β. In the WNT signaling pathway, GSK-3 inactivation stabilizes β-catenin, promoting tumor growth [39–41]. The current study showed that GAD1 overexpression in U87 and T98G cells significantly lowered p-GSK3β (ser9) and β-catenin levels, while GAD1 knockdown increased p-GSK3β in LN229 and SNB19 cells (Fig. 4B, C).

Cyclin D1 is vital for cell cycle control, particularly in the G1 to S phase transition, and promotes tumor formation [42, 43]. MMP9, part of the MMP family, aids cancer cell invasion [44, 45]. Studies have reported that β-catenin regulates Cyclin D1 and MMP9. β-catenin activates the transcription of cyclin D1 through TCF-binding sites within the promoter to boost colon cancer cell growth [46]. Hexokinase 2 enhances Wnt/β-catenin signaling, increases the levels of β-catenin, c-myc, and Cyclin D, thereby accelerating ovarian cancer proliferation [47]. β-catenin regulates the proliferation of hepatocytes by directly transcribing and controlling the expression of cyclin D1 and the transforming growth factor-α [48]. Moesin boosts hepatocellular carcinoma cell migration and invasion by activating the β-catenin/MMP9 axis [49]. IL-32 activates AKT, β-catenin, and HIF-1α, leading to the secretion of IL-8, VEGF, MMP2, and MMP9, which contribute to gastric cancer metastasis [50]. In this study, we found that β-catenin is negatively associated with GAD1 (Fig. 1D). GAD1 overexpression significantly reduced p-GSK3β and β-catenin levels, inhibiting Cyclin D1 and MMP9 (Fig. 4B, C), which in turn suppressed GBM cell proliferation, induced cell cycle arrest, and decreased migration and invasion (Fig. 2). Conversely, GAD1 knockdown increased p-GSK3β, β-catenin, Cyclin D1, and MMP9 (Fig. 4B, C), thereby promoting glioblastoma progression (Fig. 3).

AR-A014418 is an ATP competitive and selective inhibitor of GSK3β [51], inhibits the growth of pancreatic cancer cells in a dose-dependent manner by suppressing Notch 1 expression mediated by GSK-3 [52]. AR-A014418 inhibits GSK3β, thereby inducing apoptosis, reducing survival and proliferation in glioblastoma cells, while enhancing sensitivity to temozolomide and radiation [27]. This study showed that AR-A014418 inhibited glioblastoma cell proliferation, cell cycle progression, and invasion driven by GAD1 knockdown, as well as lowered the levels of p-GSK3β, β-catenin, MMP9, and Cyclin D1, but not GAD1 (Fig. 5), suggesting the regulatory role of GAD1 in the progression of GBM.

Zebrafish exhibit a 70% genomic similarity with humans and are advantageous for tumor transplantation and monitoring tumor invasion within the body, rendering them ideal models for cancer research [53, 54]. Studies have indicated that the dissemination frequency of circulating tumor cells (CTCs) clusters is lower than that of individual CTCs in zebrafish breast cancer model, and CTCs-clusters exhibit reduced invasive ability [55]. Additionally, the zebrafish xenograft model has been utilized to study glioma. Glioma stem cells (GSCs) derived from the U87 cell line were injected into the yolk sac of zebrafish, and it was observed that VEGF receptor tyrosine kinase inhibitors significantly inhibited angiogenesis induced by the xenografted U87 GSCs [56]. In the present study, the establishment of zebrafish xenograft model revealed that overexpression of GAD1 inhibits tumor proliferation and enhances the survival rate of zebrafish, whereas GAD1 knockdown produces the opposite effect (Fig. 6). These findings further substantiate the utility of zebrafish as an exemplary model for investigating glioma pathogenesis.

This study demonstrates that GAD1 influences glioblastoma progression by regulating MMP9 and Cyclin D1 via the p-GSK3β/β-catenin pathway. However, the work is subject to certain limitations. First, the precise mechanism by which GAD1 regulates p-GSK3β (Ser9) remains unclear. To address this, co-immunoprecipitation (Co‑IP) will be performed using a GAD1 antibody on glioma lysates in order to identify its direct binding partners in subsequent investigations. Second, while zebrafish represent a valuable xenograft model, their considerable evolutionary distance from humans presents a limitation. Therefore, future research could incorporate orthotopic intracranial transplantation experiments in nude mice for further validation.

In conclusion, the present study elucidates that GAD1 is downregulated in glioblastoma and serves as a predictor of poor prognosis. GAD1 inhibits the p-GSK3β/β-catenin signaling pathway, thereby reducing the expression of MMP9 and Cyclin D1, which in turn suppresses glioblastoma progression. These findings propose that GAD1 may act as a novel tumor suppressor gene, offering a potential therapeutic target for glioblastoma treatment.

## Materials and methods

### Cell lines and cell culture

The glioma cell lines (U87, U251, U373, SNB19, SW1783, SF295, A172, LN229, T98G) and immortalized normal human astrocytes (NHA) were previously described [57]. All the cells were cultured in DMEM with 10% FBS at 37 °C in a humidified 5% CO_2_ incubator, identified by STR and were mycoplasma-free. GABA (Cat# S4700) and the GSK3β inhibitor AR-A014418 (Cat# S7435) were obtained from Selleck Chemicals (Houston, Texas, USA), with AR-A014418 dissolved in DMSO and GABA in deionized water.

### Stable cell lines establishment

For stable cell lines generation, four lentiviruses (LV8N, GAD1, shNC, and shGAD1) were procured from GenePharma Co (Suzhou, China). The LV8N lentivirus was utilized to establish control cells without modifying GAD1 levels, whereas the GAD1 lentivirus, which expresses the transcript variant GAD67 representing the full-length and predominant form of GAD1, was employed to achieve GAD1 overexpression. The shNC lentivirus (5’-TTCTCCGAACGTGTCACGT-3’) functioned as a control without influencing GAD1 expression, while the shGAD1 lentivirus (5’-GCCAGACAAGCAGTATGATGT-3’) was applied to downregulate GAD1 expression. U87 and T98G cells were infected with LV8N or GAD1, whereas LN229 and SNB19 cells were infected with shNC or shGAD1. One day post-infection, the cells were cultured in a medium containing 2 μg/mL puromycin for two weeks to select for stably infected cells, which were subsequently confirmed via Western blot analysis.

### Western blot

Cells were washed twice with ice-cold PBS and then lysed in RIPA buffer (Beyotime, Shanghai, China) mixed with PMSF (100×, Beyotime), protease inhibitor cocktail (100×, Beyotime), and phosphatase inhibitor PhosSTOP (Roche, Basel, Switzerland) according to the method mentioned before [58, 59]. Proteins (20 μg per sample) were separated via 10% SDS-PAGE and transferred to PVDF membranes. Membranes were incubated with primary antibody overnight at 4 °C, then with HRP-conjugated goat anti-rabbit IgG (511203, ZENBIO, Chengdu, China) for 1 h at room temperature. Signals were detected using a chemiluminescent imaging system (eBlot Touch Imager, China). Primary antibodies used were anti-MMP9 (AF5234, Beyotime), anti-β-catenin (51067-2-AP, Proteintech, Wuhan, China), anti-GAD1 (R22798, ZENBIO), anti-phospho-GSK3β(Ser9) (R22943, ZENBIO), anti-GSK3β (22104-1-AP, Proteintech), anti-Cyclin D1 (R380999, ZENBIO), anti-GAPDH (R380626, ZENBIO).

### Cell proliferation assay

Cell proliferation was assessed with the Cell Counting Kit-8 (CCK-8, APExBIO, Houston, Texas, USA). Cells were seeded at 1000 cells/100 μl per well in 96-well plates and cultured for specified days. After adding 10 μl of CCK-8 reagent, the cells were incubated for 2 h at 37 °C, and absorbance at 450 nm was measured using a microplate reader (Cytation 5, Bio Tek, USA).

### Wound healing assay

For migration analysis, cells were grown in 6-well plates to 90% confluence, scratched with sterile pipette tips, and cultured in serum-free medium. Images were captured at 0 and 24 h to measure wound width, and migration efficiency was calculated as: [(wound width at 0 h - wound width at 24 h)/wound width at 0 h] × 100%.

### Colony formation assay

Cells were seeded in 6-well plates at 400 cells per well and cultured for 10–14 days. They were fixed with 4% paraformaldehyde and stained with 0.1% crystal violet. Colonies in each well were counted for the analysis of colony formation activity.

### Cell invasion assay

Transwell chambers with 8 μm pores were coated with Matrigel (KeyGEN, Nanjing, China). Cells (3 × 10^4^ per insert) in serum-free DMEM were added to the upper chamber, and DMEM with 10% serum in the lower chamber. After 24 h, non-invaded cells were removed using cotton swabs, and the invaded cells were fixed, stained with 0.1% crystal violet, and photographed. Then, the stained cells were dissolved in 5% SDS and read by spectrophotometer (Cytation 5, Bio Tek) for OD570.

### Cell cycle analysis

Cells were collected and fixed in 75% ice-cold ethanol overnight at 4 °C, treated with 50 μg/ml RNaseA for 30 min at 37 °C, and stained with 50 μg/ml propidium iodide for 30 min at 4 °C in the dark. Cell cycle distribution was analyzed via flow cytometry (Cytoflex, Beckman, USA).

### Animal care and handling

The adult AB zebrafish were kept in the Soochow University Zebrafish Facility at 28.5 °C with controlled lighting (9 am to 11 pm). They were fed with brine shrimp in the morning and afternoon, and dry flake food at noon. Zebrafish embryos were incubated in E3 buffer at 28 °C until use.

### Zebrafish xenotransplantation

To perform the zebrafish xenotransplantation experiment, we selected T98G-LV8N and T98G-GAD1cells, as well as SNB19-shNC and SNB19-shGAD1 cells. Glioma cells were trypsinized, made into a single-cell suspension, and stained with the cell membrane red fluorescent probe Dil (Beyotime) for 20 min. Using a fluorescence microscope, it was observed that all the stained cells emitted orange-red fluorescence. Then the stained cells were diluted to 7.5 × 10^6^ cells/ml. Wild-type zebrafish larvae at 2 days post-fertilization (dpf) were anesthetized with tricaine and divided into four groups randomly (T98G-LV8N, T98G-GAD1, SNB19-shNC, SNB19-shGAD1), with 20 zebrafish per group. Approximately 150 fluorescent cells were injected into the central of yolk sac region using a pneumatic microinjection pump with an injection needle.

After microinjection, larvae were examined using a Nikon SWZ18 fluorescent microscope to track tumor progression. Both bright-field and fluorescent images were captured at 2 hpi (hours post-injection) and 48 hpi with a Nikon DS-Ri2 digital camera controlled with NIS-Elements D 5.20 Express software. The fluorescence emitted by the tumor masses within the larvae was measured using ImageJ, and results were plotted as histograms. Zebrafish survival times were recorded for survival curves. Personnel performing the analysis of in vivo tumor growth and survival curves in zebrafish were blinded to group allocation. All experimental procedures involving zebrafish were conducted in compliance with the ethical guidelines approved by the Animal Ethics Committee of Soochow University.

### Statistical analysis

All statistical analyses were performed using GraphPad Prism 8.0. Data are presented as mean ± SD with individual data points, derived from at least three biological replicates. Sample sizes were determined a priori based on established practices in prior, comparable studies, and are indicated in the respective figures. For comparisons between two groups, unpaired Welch’s *t*-test was applied. For comparisons involving multiple groups, one-way ANOVA or two-way ANOVA was used, as appropriate. Survival curves were generated by the Kaplan–Meier method, and the differences between groups were assessed using the log-rank test. The correlation between β-catenin and GAD1 expression levels was evaluated using Pearson’s correlation analysis. Statistical significance was defined as *p < 0.05, **p < 0.01, ***p < 0.001.

## Supplementary information

Original Data of Western blots

## Data Availability

The data that support the findings of this study are available from the corresponding author upon reasonable request.
