# Supplementary material for: Glutamate decarboxylase 1 (GAD1) suppresses the progression of glioblastoma through GSK3β/β-catenin pathway
Source: Cell Death Discov. 2026 Mar 17;12:132. doi: 10.1038/s41420-026-02997-0 (PMC13039808; doi:10.1038/s41420-026-02997-0)
Supplement: Supplementary file 1 — Original Data of Western blots [file 41420_2026_2997_MOESM1_ESM.pdf]

Figure S1

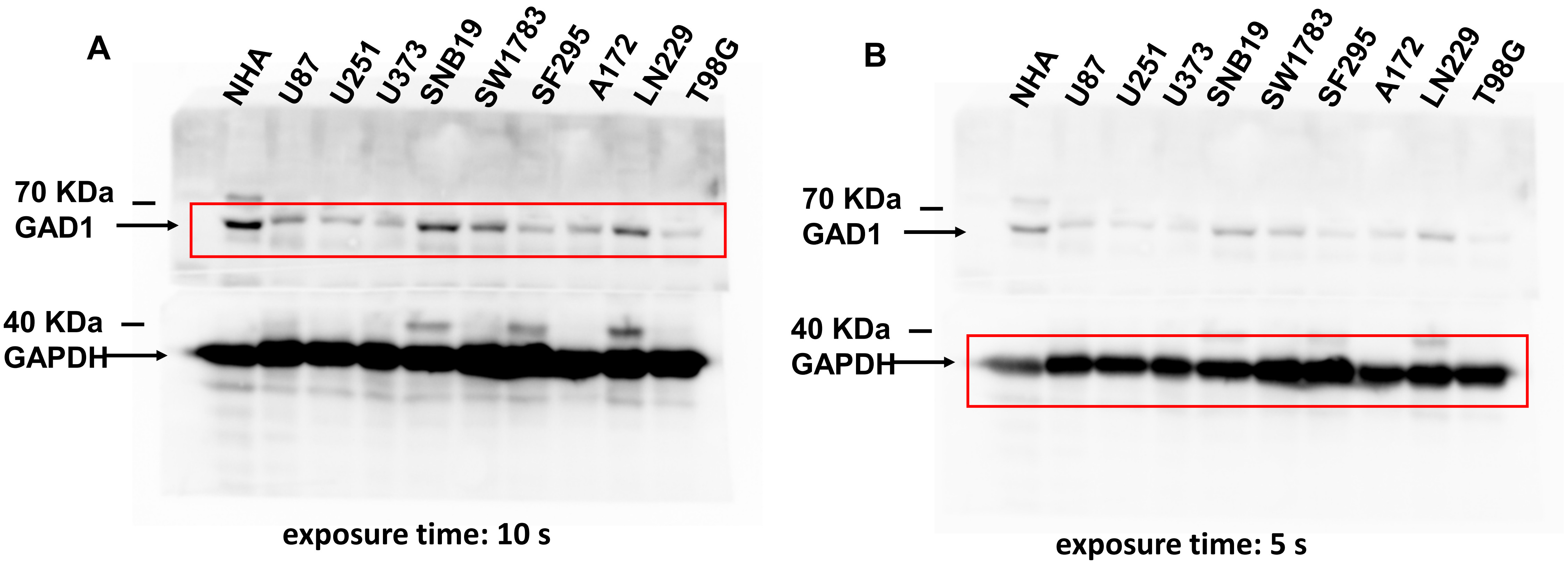

Figure S1. The original images of Western blot related to Figure 1A. The bands of GAD1 (A) and GAPDH (B) within the red box are shown in Figure 1A.

Figure S2

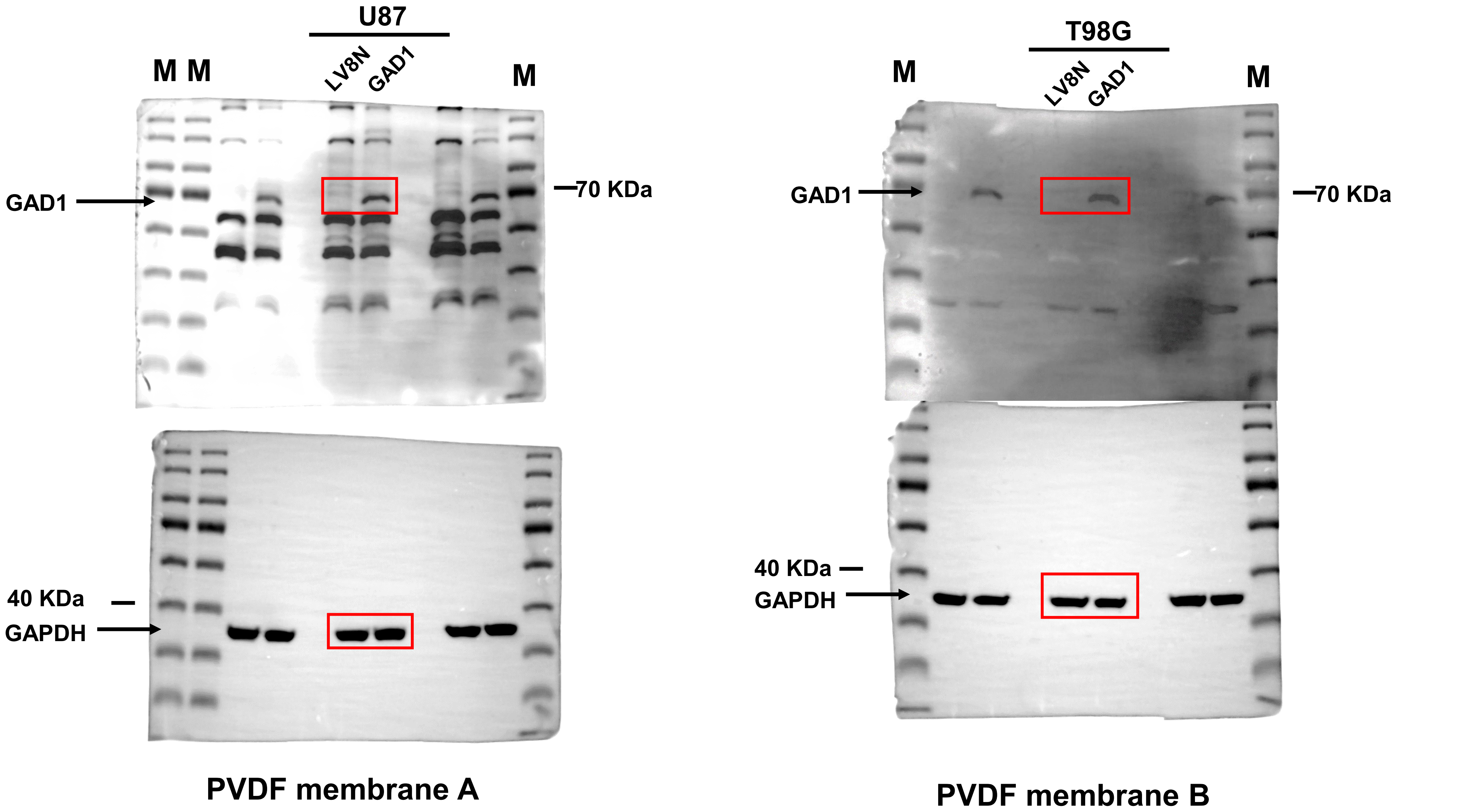

Figure S2. The original images of Western blot related to Figure 2A. The bands within the red boxes in PVDF membrane A are related to U87 cells, and the bands within the red boxes in PVDF membrane B are related to T98G cells, as shown in Figure 2A.

Figure S3

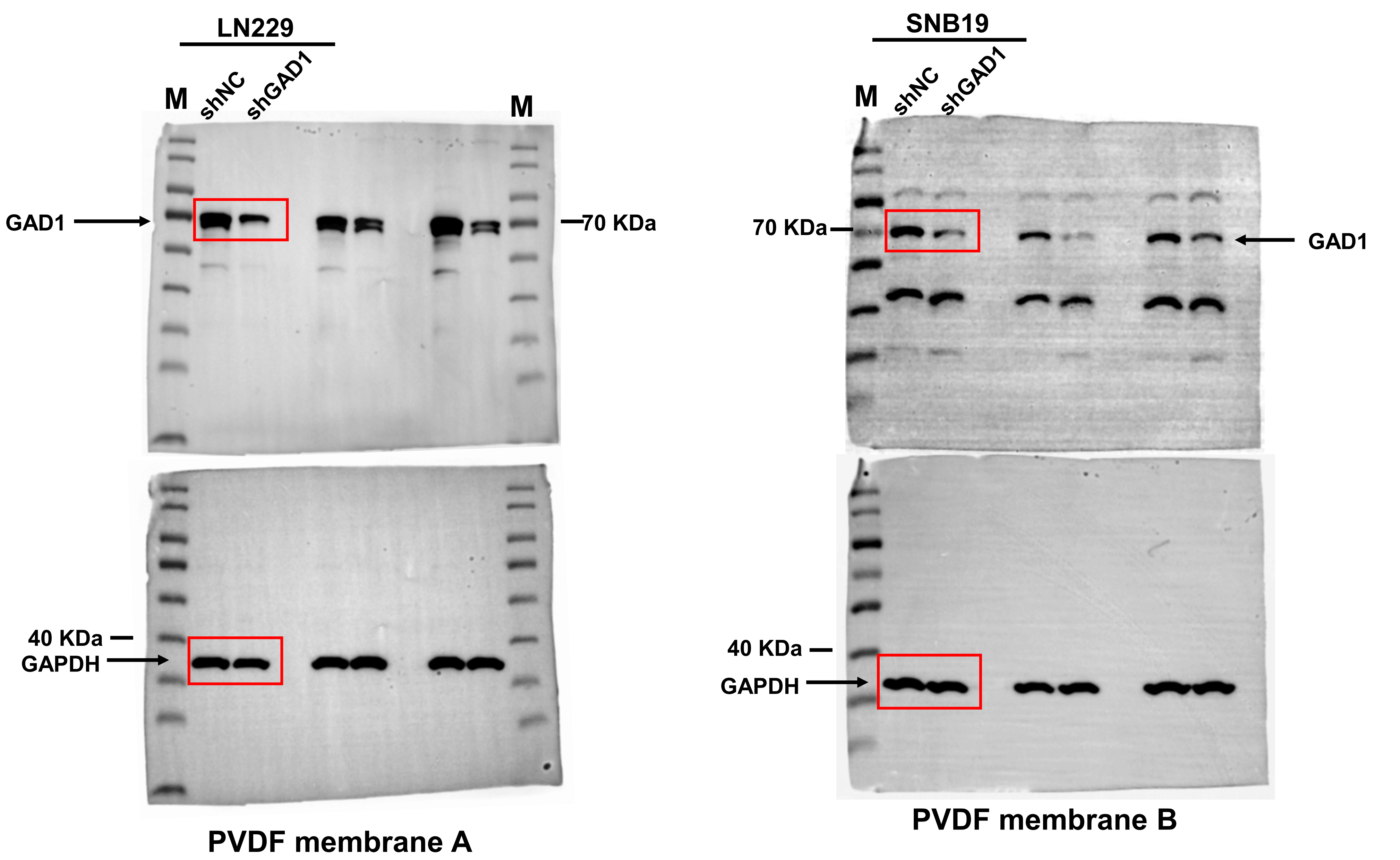

Figure S3. The original images of Western blot related to Figure 3A. The bands within the red boxes in PVDF membrane A are related to LN229 cells, and the bands within the red boxes in PVDF membrane B are related to SNB19 cells, as shown in Figure 3A.

Figure S4

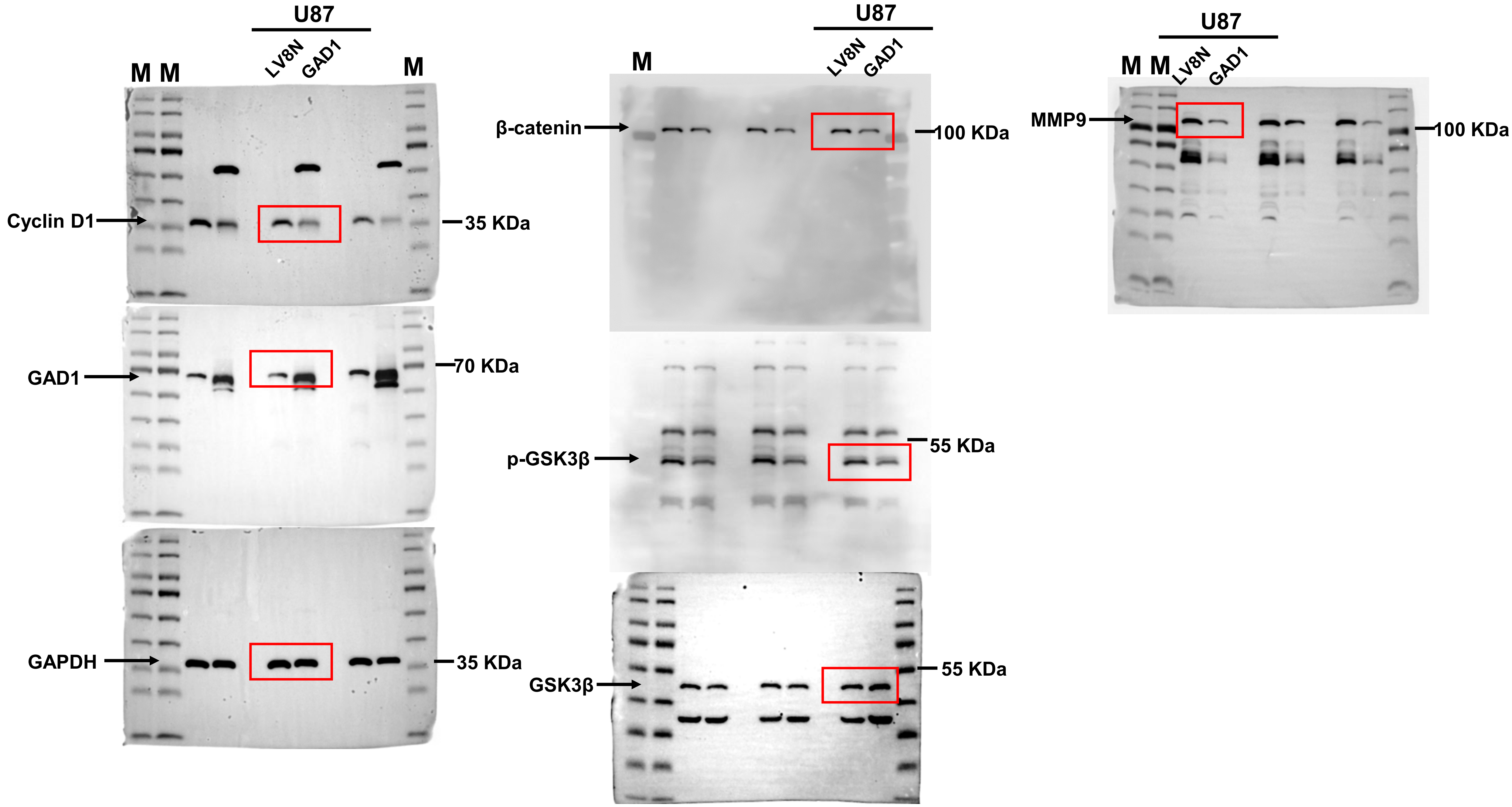

Figure S4. The original images of Western blot related to the U87cells in Figure 4B. The bands of U87-LV8N and U87-GAD1 within the red boxes are shown in Figure 4B.

Figure S5

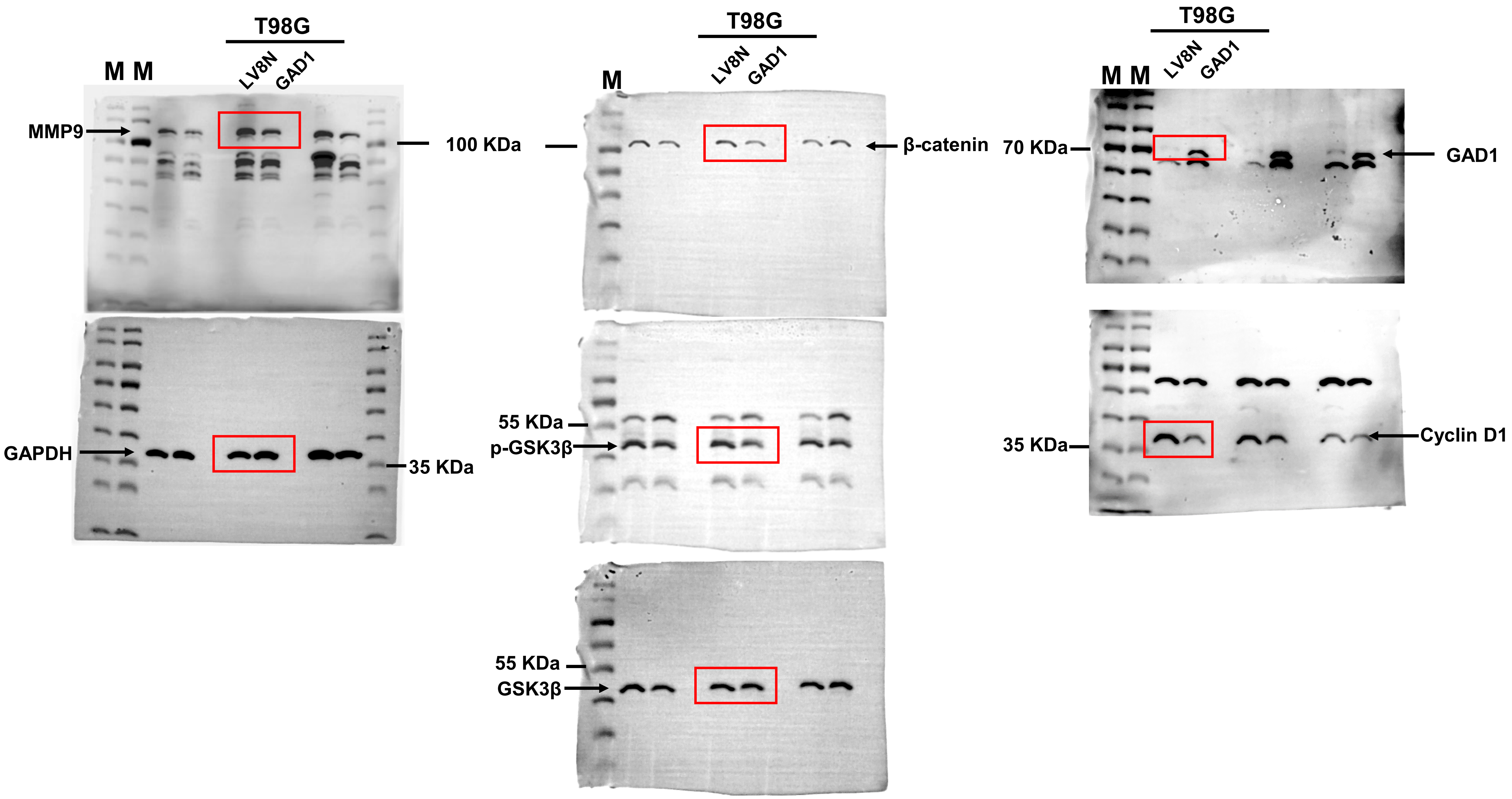

Figure S5. The original images of Western blot related to the T98G cells in Figure 4B. The bands of T98G-LV8N and T98G-GAD1 within the red boxes are shown in Figure 4B.

Figure S6

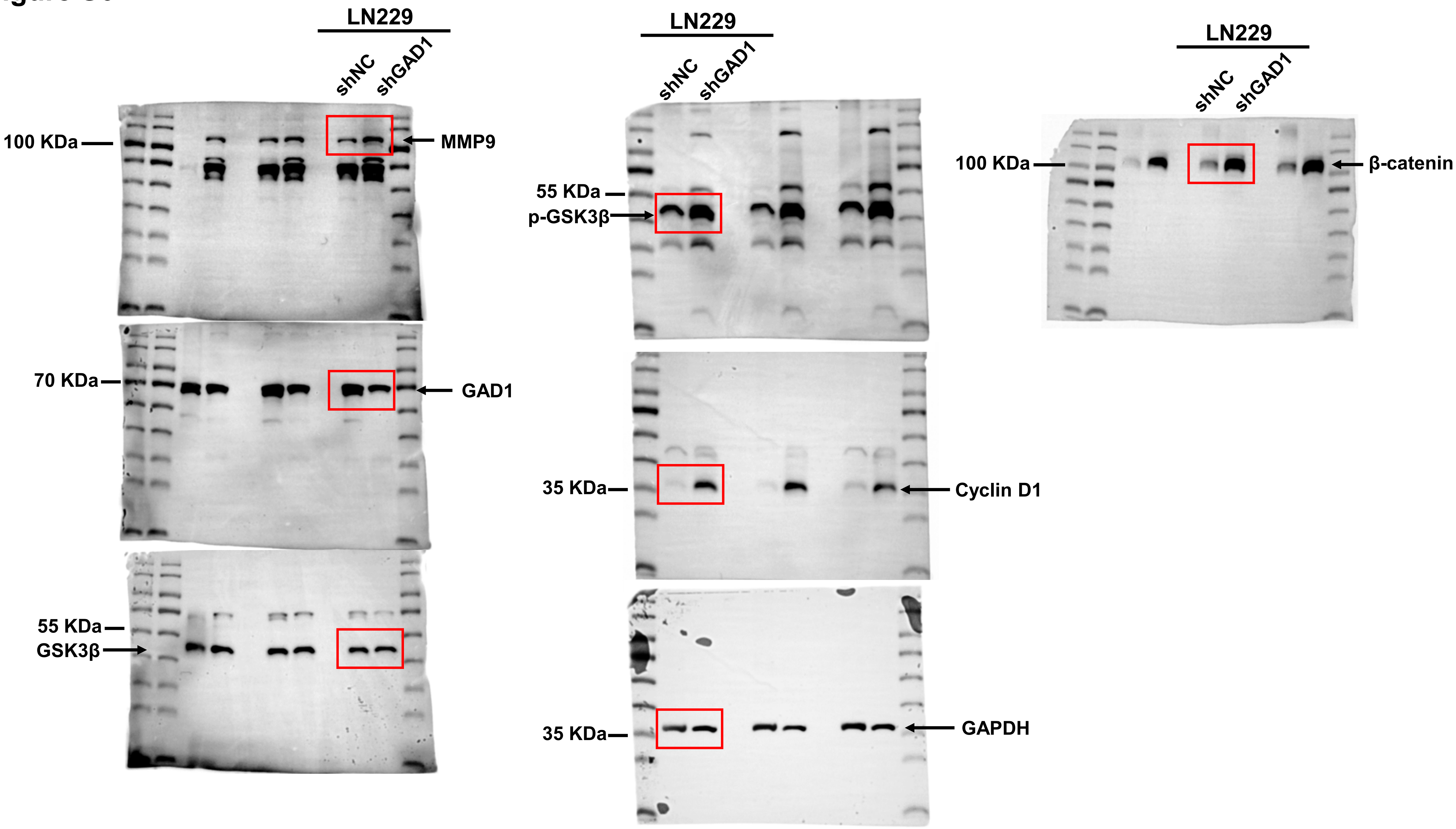

Figure S6. The original images of Western blot related to the LN229 cells in Figure 4B. The bands of LN229-shNC and LN229-shGAD1 within the red boxes are shown in Figure 4B.

Figure S7

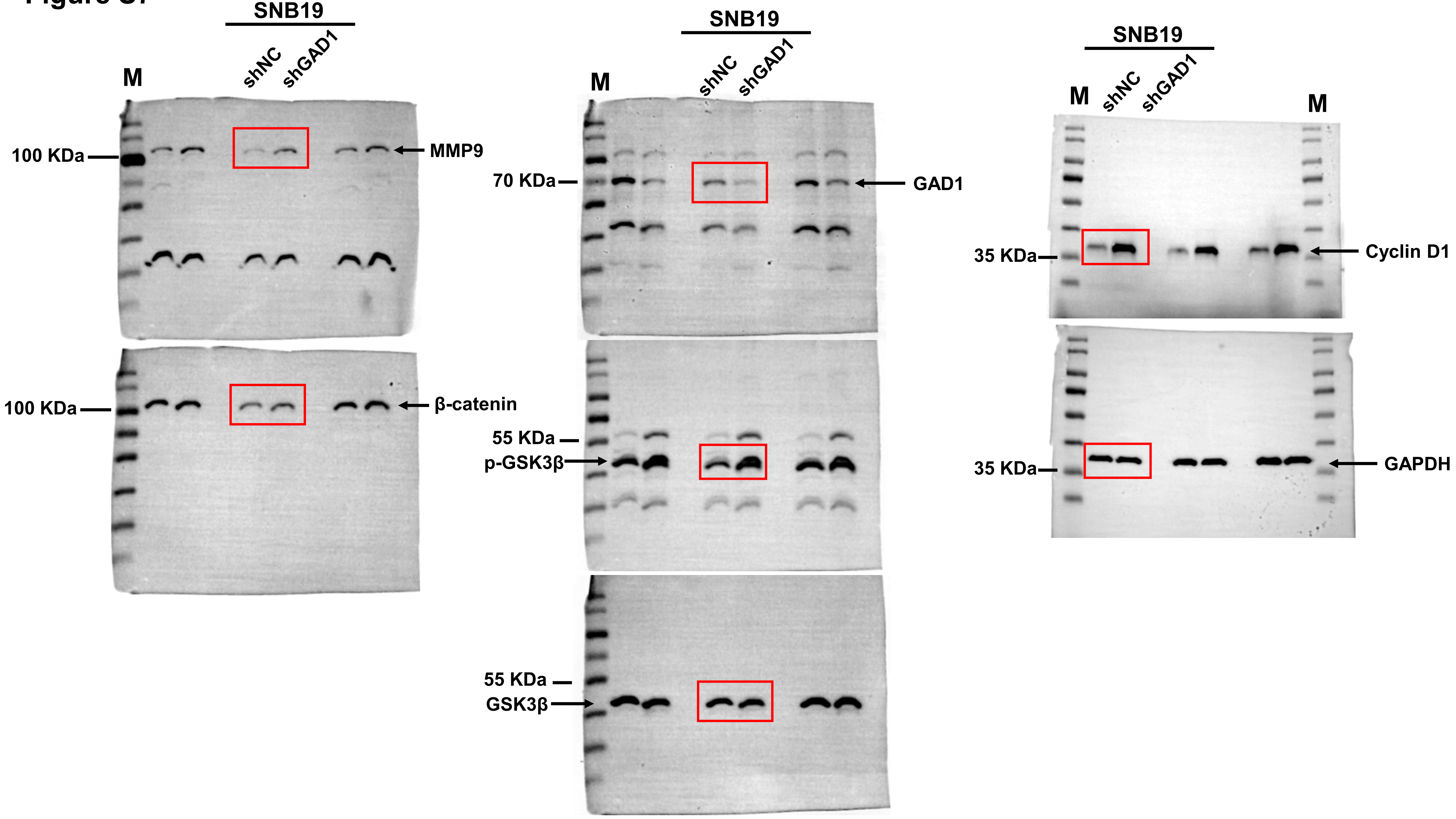

Figure S7. The original images of Western blot related to the SNB19 cells in Figure 4B. The bands of SNB19-shNC and SNB19-shGAD1 within the red boxes are shown in Figure 4B.

Figure S8

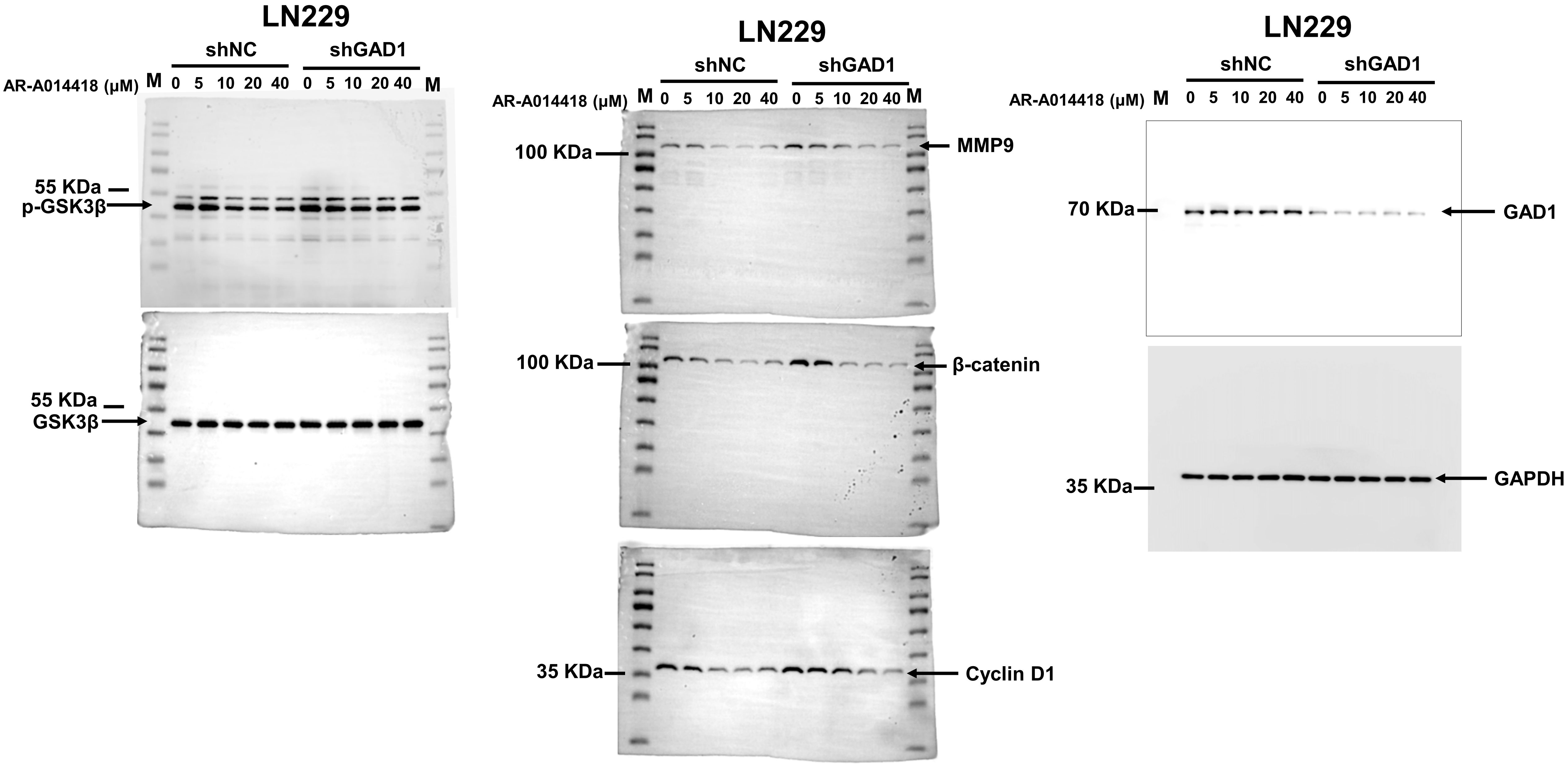

Figure S8. The original images of Western blot related to the LN229 cells in Figure 5B. The bands of LN229-shNC and LN229-shGAD1 are shown in Figure 5B.

Figure S9

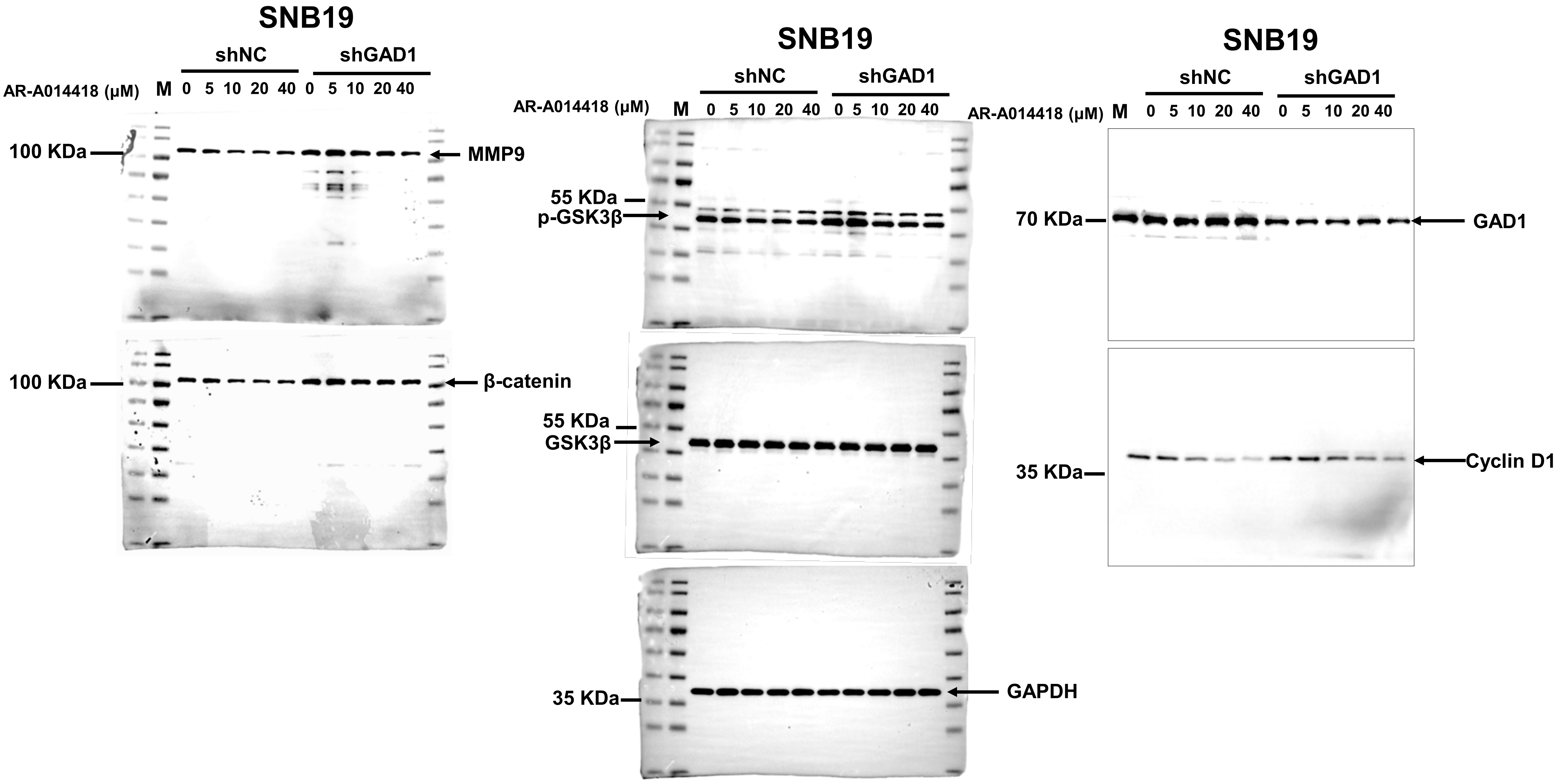

Figure S9. The original images of Western blot related to the SNB19 cells in Figure 5B. The bands of SNB19-shNC and SNB19-shGAD1 are shown in Figure 5B.
